# Supplementary material for: Heterogeneously integrated lithium tantalate-on-silicon nitride modulators for high-speed communications
Source: Nat Commun. 2026 Feb 28;17:3314. doi: 10.1038/s41467-026-69769-3 (PMC13066381; doi:10.1038/s41467-026-69769-3)
Supplement: Supplementary file 1 — Supplementary Information [file 41467_2026_69769_MOESM1_ESM.pdf]

Supplementary Information for: Heterogeneously integrated lithium tantalate-on-silicon nitride modulators for high-speed communications

Jiachen Cai,[1,2,*] Alexander Kotz,[3,*] Hugo Larocque,[2,*] Chengli Wang,[1,2] Xinru Ji,[2] Junyin Zhang,[2] Daniel Drayss,[3] Jiale Sun,[2] Shuhang Zheng,[2] Xin Ou,[1,†] Christian Koos,[3,‡] and Tobias J. Kippenberg[2,4,§]

[1]*State Key Laboratory of Materials for Integrated Circuits, Shanghai Institute of Microsystem and Information Technology, Chinese Academy of Sciences, Shanghai, China*
[2]*Institute of Physics, Swiss Federal Institute of Technology Lausanne (EPFL), CH-1015 Lausanne, Switzerland*
[3]*Institute of Photonics and Quantum Electronics (IPQ), Karlsruhe Institute of Technology (KIT), 76131 Karlsruhe, Germany*
[4]*Institute of Electrical and Micro engineering, Swiss Federal Institute of Technology, Lausanne (EPFL), CH-1015 Lausanne, Switzerland*

Contents

Supplementary Note 1. Photonic Damascene process for integrated silicon nitride waveguides 2

Supplementary Note 2. Simulations for silicon nitride-lithium tantalate Mach-Zehnder modulators (MZMs) 3
A. Trade-off between $V_\pi \cdot L$ and optical loss 3
B. Wave velocity matching 4

Supplementary Note 3. Wafer-level characterization 6
A. Thin film uniformity 6
B. Device uniformity 7

Supplementary Note 4. Simulations for tapered mode transitions 9

Supplementary Note 5. Microwave transmission and modulation efficiency 10

Supplementary Note 6. Photonic package for DC-bias drift measurements 11

Supplementary Note 7. Digital signal processing at the receiver for silicon nitride-lithium tantalate modulator-based communication experiments 11

Supplementary Note 8. Extended signal quality and energy efficiency analysis for the high-speed data transmission experiments 12
A. Normalized generalized mutual information (NGMI) and error vector magnitude (EVM) 12
B. Energy dissipation 13

Supplementary Note 9. Comparison to the state-of-the-art 14

---

* These authors contributed equally.
† ouxin@mail.sim.ac.cn
‡ christian.koos@kit.edu
§ tobias.kippenberg@epfl.ch

Supplementary Note 1. Photonic Damascene process for integrated silicon nitride waveguides

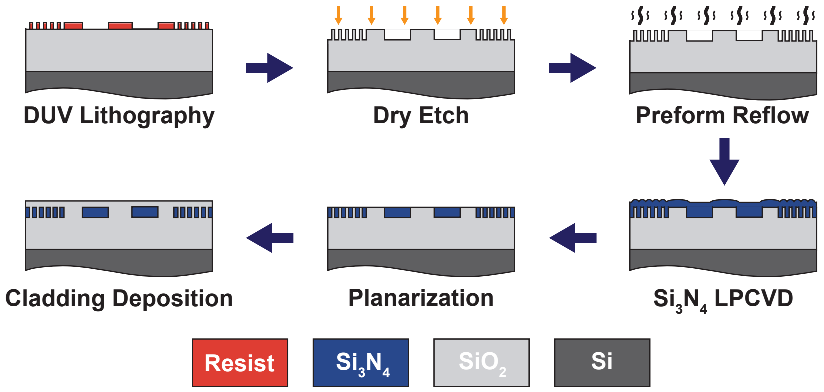

Supplementary Figure 1. **Wafer-scale photonic Damascene process for low-loss** Si_3N_4 **photonic integrated circuits.** The process flow includes deep ultraviolet (DUV) stepper lithography, fluoride dry etching, preform reflow, high-density Si_3N_4 deposition, chemical mechanical polishing (CMP), and cladding deposition.

The process begins with a standard wet oxide substrate (4 µm SiO_2/525 µm Silicon). The waveguide and filler patterns are defined on the top silica using an ASML PAS 5500/350C DUV stepper with a 248 nm wavelength light source. The micron-order filler patterns are designed as quasi-intersecting line structures to mitigate the tensile stress associated with high density Si_3N_4 deposition. These patterns are then transferred into the underlying wet oxide via fluorine-based dry etching to a depth of 500 nm. A subsequent high-temperature annealing step at 1250°C is performed to promote oxide reflow and improve sidewall smoothness. After annealing, a 700 nm-thick Si_3N_4 layer is deposited into the trenches using low pressure chemical vapor deposition (LPCVD). A dedicated-adjusted CMP process is implemented to remove the redundant material outside the trench, as well as enabling ultralow surface roughness on the top surface of Si_3N_4 waveguides. A 1 µm thick oxide interlayer is deposited by LPCVD, after which an additional 1200°C annealing is conducted to eliminate absorption losses associated with hydrogen impurities. Finally, a second CMP step is adopted to polish the interlayer to a thickness of 50 nm so as to remove the residual topography due to the Damascene process, as well as ensuring sub-nanometer surface roughness for subsequent wafer bonding of lithium tantalate on Damascene silicon nitride wafers.

Supplementary Note 2. Simulations for silicon nitride-lithium tantalate Mach-Zehnder modulators (MZMs)

A. Trade-off between $V_\pi \cdot L$ and optical loss

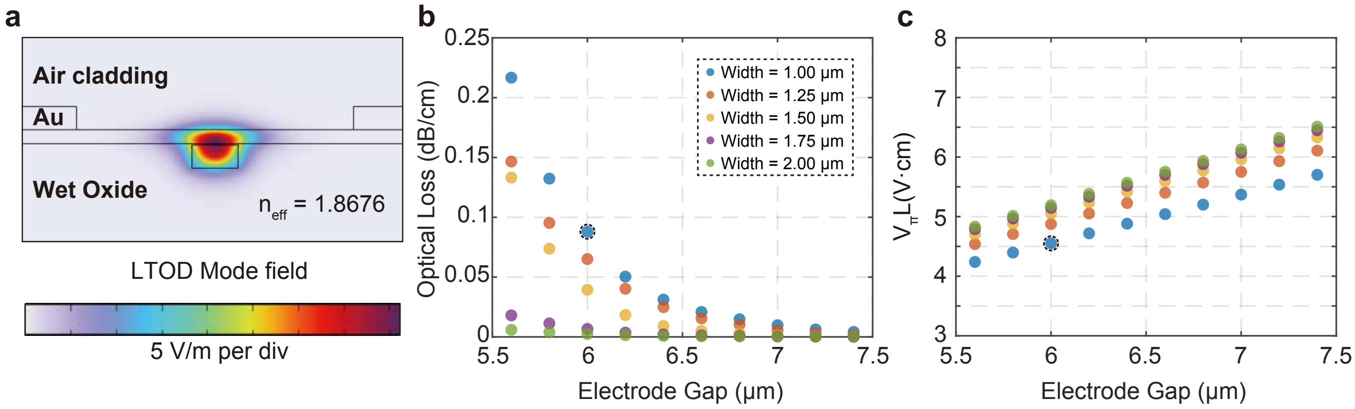

Supplementary Figure 2. Static field simulation of silicon nitride-lithium tantalate Mach-Zehnder modulators. (a) Simulated optical mode field in the cross-section of the hybrid Si_3N_4-$LiTaO_3$ waveguide (LTOD). (b) Calculated optical loss and (c) $V_\pi \cdot L$ versus different electrode gap values and Si_3N_4 waveguide widths. The hollow circles mark the estimated performance for the fabricated Si_3N_4-$LiTaO_3$ modulators reported in this work, with a waveguide width of 1 μm and an electrode gap of 6 μm.

Optical simulations (COMSOL multiphysics) were first employed to calculate the mode profiles in Si_3N_4-$LiTaO_3$ hybrid waveguides. Given the fabrication process from the main text and **Supplementary Note 1**, the thickness of $LiTaO_3$ and Si_3N_4 is chosen to be 300 nm and 500 nm, respectively. Supplementary Figure 2(a) represents the electric field distribution of the hybrid optical mode. The confined mode is divided roughly equally between the Si_3N_4 ridge waveguide and the lithium tantalate thin film. As stated in the Discussion part of the main text, alternative mode distributions are feasible by altering the film thickness, but it involves changes in the preparation of the lithium tantalate wafer, such as adjusting the ion-implantation dose and annealing temperature during the corresponding smart-cut process [1, 2]. Furthermore, modifying the film thickness also introduces a performance trade-off between actuation voltage and optical insertion loss. Efficient electro-optic (EO) modulation can be achieved by adopting a close enough electrode-to-waveguide spacing at the expense of larger optical absorption. Here, the device geometry is well optimized using finite element simulations. Assuming that the propagation mode is transverse electric (TE), the estimated loss related to various electrode gaps can be extracted from the imaginary part of the effective mode index (n_{eff}),

$$\alpha = 0.1 \times |\log_{10}(e^{-\frac{4\pi}{\lambda}\cdot \text{Im}(n_{\text{eff}})})| \quad (\text{dB/cm}), \tag{1}$$

where λ represents the wavelength of the selected mode. Supplementary Figure 2(b) shows how the electrode gap affects transmission loss for various Si_3N_4 waveguide widths.

To model the EO performance of the hybrid $LiTaO_3$-on-Si_3N_4 modulator, we first consider the effect of a perturbation, $\boldsymbol{\Delta\epsilon}$, on the dielectric permittivity tensor, $\boldsymbol{\epsilon}$, of the underlying waveguide. From perturbation theory [3], the resulting first order frequency shift of an eigenfunction $\vec{E}_n$ satisfying the optical wave equation is defined as

$$\Delta\omega_n = -\frac{1}{2}\omega_n \frac{\int dV \left(\boldsymbol{\Delta\epsilon}\, \vec{E}_n\right) \cdot \vec{E}_n^*}{\int dV \left(\boldsymbol{\epsilon} \vec{E}_n\right) \cdot \vec{E}_n^*}, \tag{2}$$

For a guided mode propagating in a waveguide of length L, this perturbation results in a propagation constant change $\Delta\beta_n = \Delta\omega_n/c$. For a pair of such waveguides arranged in a push-pull MZM configuration, the resulting offset between the two becomes $\Delta\phi = 2\Delta\beta_n L$. By setting the condition for half-wave modulation, $\Delta\phi = \pm\pi$, the general expression

for the half-wave voltage-length product ($V_\pi \cdot L$) becomes:

$$V_\pi \cdot L = \frac{V\pi}{2\Delta\beta_n} = \frac{V\pi c}{2\Delta\omega_n} = \frac{V\pi c}{\omega_n}\frac{\int dA\left(\boldsymbol{\epsilon}\vec{E}_n\right)\cdot\vec{E}_n^*}{\int dA\left(\boldsymbol{\Delta\epsilon}\,\vec{E}_n\right)\cdot\vec{E}_n^*} = \frac{V\lambda}{2n_{\text{eff}}}\frac{\int dA\left(\boldsymbol{\epsilon}\vec{E}_n\right)\cdot\vec{E}_n^*}{\int dA\left(\boldsymbol{\Delta\epsilon}\,\vec{E}_n\right)\cdot\vec{E}_n^*}, \tag{3}$$

where V is the applied voltage, λ is the optical wavelength, and n_{eff} is the effective index of the eigenmode. Given the guided nature of the mode, the original volume integral reduces to a surface one over the waveguide cross-section. For a Pockels material perturbed by a DC electric field $\vec{E}_{\text{DC}}$, the change in the permittivity tensor is given by $\boldsymbol{\Delta\epsilon}^{-1} = \boldsymbol{r}\vec{E}_{\text{DC}}$, where $\boldsymbol{r}$ is the Pockels tensor. For the case of $LiTaO_3$ enclosed by electrodes applying a DC field oriented along the crystal's extraordinary axis, the perturbative term in Eqs. (2, 3) to first order becomes [4]:

$$\left(\boldsymbol{\Delta\epsilon}\,\vec{E}_n\right)\cdot\vec{E}_n^* = \left[r_{13}\epsilon_{\text{o}}^2\left(|E_{n,1}|^2+|E_{n,2}|^2\right) + r_{33}\epsilon_{\text{e}}^2|E_{n,3}|^2\right]E_{\text{DC}}. \tag{4}$$

For a Si_3N_4-$LiTaO_3$ hybrid modulator, the only Pockels material is $LiTaO_3$, thus allowing us to only consider this material while integrating over the permittivity perturbation. Therefore, the corresponding $V_\pi \cdot L$ value of the modulator becomes:

$$V_\pi \cdot L = \frac{V\lambda}{2n_{\text{eff}}}\frac{\int dA\,\epsilon_{11}(\mathbf{r})\,|E_{n,1}(\mathbf{r})|^2 + \epsilon_{22}(\mathbf{r})|E_{n,2}(\mathbf{r})|^2 + \epsilon_{33}(\mathbf{r})|E_{n,3}(\mathbf{r})|^2}{\int_{\text{LiTaO}_3} dA\,E_{\text{DC}}(\mathbf{r})\left[r_{13}\epsilon_o^2\left(|E_{n,1}(\mathbf{r})|^2 + |E_{n,2}(\mathbf{r})|^2\right) + r_{33}\epsilon_e^2|E_{n,3}(\mathbf{r})|^2\right]}, \tag{5}$$

which, when applied to monolithic thin film or bulk Pockels modulators, results in a simplified expression such as the one provided in [5]. Given the unpatterned $LiTaO_3$ layer and the electrodes lying directly on it, $E_{\text{DC}}(\mathbf{r})/V$ roughly corresponds to the modulator's electrode gap size. Supplementary Table 1 provides the parameter values we use to model the $V_\pi \cdot L$ of our modulator.

Supplementary Table 1. **Parameter values used to model electro-optic performance.**

Parameter	Description	Value
λ	Optical Wavelength	1.55 µm
n_{eff}	Effective refractive index	1.828
ϵ_{Air}	Relative permittivity of air (defines ϵ_{11}, ϵ_{22}, ϵ_{33})	$(1)^2$
ϵ_{SiO2}	Relative permittivity of SiO_2 (defines ϵ_{11}, ϵ_{22}, ϵ_{33})	$(1.444)^2$
ϵ_{SiN}	Relative permittivity of Si_3N_4 (defines ϵ_{11}, ϵ_{22}, ϵ_{33})	$(1.992)^2$
ϵ_{o}	Ordinary relative permittivity of $LiTaO_3$ (defines ϵ_{11}, ϵ_{22})	$(2.123)^2$
ϵ_{e}	Extraordinary relative permittivity of $LiTaO_3$ (defines ϵ_{33})	$(2.119)^2$
r_{13}	13 component of $LiTaO_3$'s Pockels tensor	8.4 pm/V
r_{33}	33 component of $LiTaO_3$'s Pockels tensor	30.5 pm/V
$E_{\text{DC}}(\mathbf{r})/V$	DC field to applied voltage ratio	6 µm
	Si_3N_4 waveguide dimensions	1 µm× 0.5 µm
	$LiTaO_3$ film thickness	300 nm
	Interlayer oxide thickness	30 nm

As plotted in Supplementary Figure 2(c), the 1 µm-wide waveguide shows higher modulation efficiency because of a larger optical mode overlap with the lithium tantalate thin film. Based on the above analysis, we opt for a device geometry featuring a signal-ground spacing of 6 µm, metal thickness of 800 nm and a Si_3N_4 waveguide width of 1 µm to obtain a high modulation efficiency with negligible insertion loss. The slight difference between the measured $V_\pi \cdot L$ and the simulation result is attributed to our fabrication tolerances. Key factors include variations in the final Si_3N_4 thickness from the CMP non-uniformity and the deviations in the CPW electrode gap defined by the resolution of the maskless lithography.

B. Wave velocity matching

To sustain interactions between propagating microwaves and optical modes over long distances, the velocities of these two fields must be similar [6]. For a fixed optical waveguide geometry, such a requirement can be satisfied by

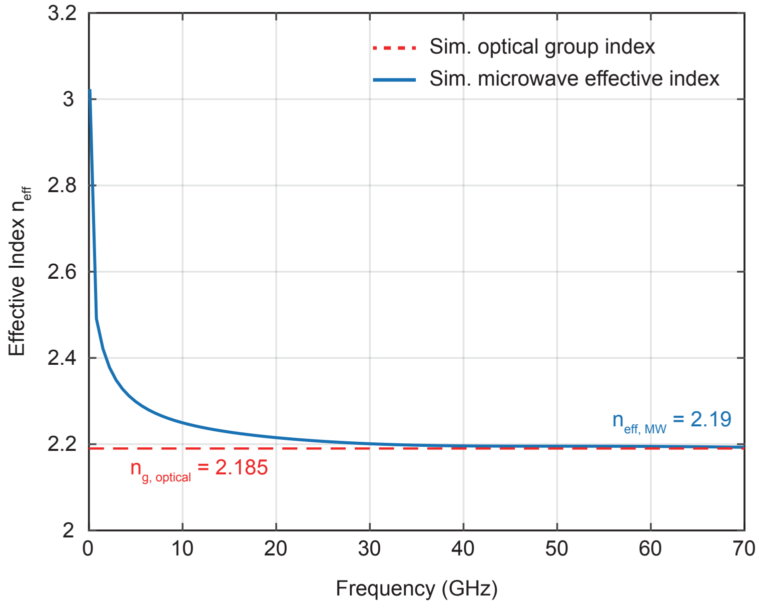

Supplementary Figure 3. Velocity mismatch between microwave and lightwave. Simulated optical group index and microwave effective index, showing a negligible disparity in the high modulation frequency range.

a suitable traveling wave electrode design. We simulate the effective refractive index ($n_{\text{eff,MW}}$) of a propagating RF field in high-speed electrodes using Ansys HFSS. The $n_{\text{eff,MW}}$ can be extracted by phase unwrapping the transmission S_{21} of coplanar waveguide (CPW) electrodes:

$$n_{\text{eff}} = c_0 \frac{\text{unwrap}(\text{ang}(S_{21}))}{2\pi\omega_{\text{RF}} L_{\text{ele}}}, \tag{6}$$

where c_0 is the speed of light in vacuum, ω_{RF} is the RF modulation frequency and L_{ele} is the length of the CPW electrodes. The group refractive index ($n_{\text{g,optical}}$) of the waveguide's optical TE mode is obtained using Ansys Lumerical Mode. Supplementary Figure 3 implies a near-perfect alignment between the optical n_{g} and the microwave n_{eff} above a modulation frequency of 40 GHz, thereby verifying the required phase-matching behavior for an Si_3N_4-$LiTaO_3$ modulator with a high EO bandwidth.

Supplementary Note 3. Wafer-level characterization

A. Thin film uniformity

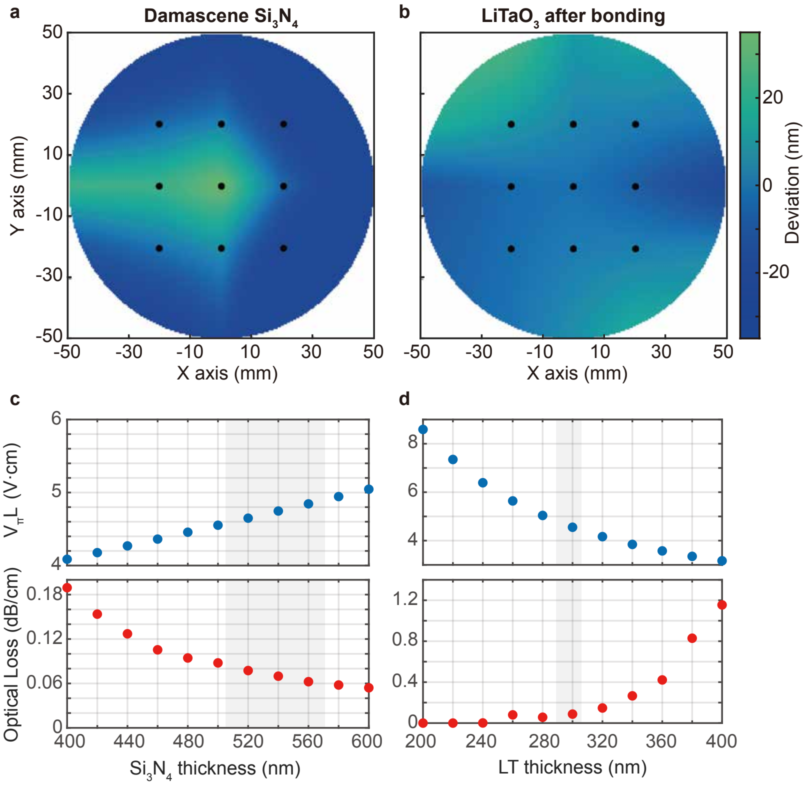

Supplementary Figure 4. Analysis of thickness variations and their impact on device performance. Thickness maps of the (a) silicon nitride layer and (b) lithium tantalate thin film. The maps are interpolated from measurements at the nine central stepper fields (marked as black dots). Simulated modulation efficiency and optical loss as a function of (c) the silicon nitride waveguide height and (d) the lithium tantalate thin film thickness. The shaded regions indicate the measured range of film thickness on the wafers.

To quantify the wafer-scale process yield of the Si_3N_4-$LiTaO_3$ platform, Supplementary Figure 4(a,b) show the measured thickness maps of the Si_3N_4 waveguide (post-Damascene process) and thin film $LiTaO_3$ (post-bonding) layers, respectively. The raw data, measured at the center of the nine stepper fields defined in Supplementary Figure 5(a) are (532 nm, 539 nm, 511 nm; 559 nm, 571 nm, 531 nm; 535 nm, 543 nm, 505 nm) for the Si_3N_4 layer and (306 nm, 300 nm, 298 nm; 293 nm, 295 nm, 289 nm; 294 nm, 298 nm, 299 nm) for the $LiTaO_3$ layer. The Si_3N_4 thickness features height variations introduced by the surface polishing step of the Damascene process. Additional CMP on the interlayer used for bonding can further modify the absolute Si_3N_4 thickness across the wafer. In contrast, the bonded $LiTaO_3$ thin film exhibits much higher uniformity, with total variations near 17 nm.

Based on the simulated results in Supplementary Figure 4(c,d), increasing the waveguide height within the measured range of 500 to 560 nm only slightly decreases optical loss due to stronger mode confinement in the Si_3N_4 core. This reduction comes at the cost of an increased V_π from 4.55 to 4.85 V. As for the $LiTaO_3$ thickness, a tens-of-nanometer variation significantly alters V_π, while a thicker $LiTaO_3$ thin film increases metal-induced absorption loss. Therefore, we identify the uniformity of the $LiTaO_3$ layer as the most critical parameter for achieving consistent device performance. The excellent uniformity provided by the ion-slicing and wafer-bonding techniques is therefore crucial for this platform. To further improve wafer-level device consistency, using a subtractive process to fabricate the Si_3N_4 waveguide [7] can mitigate dimensional changes that arise from the polishing and thermal reflow fabrication steps. However, achieving a flat surface required for high-yield bonding is considerably more complex with this subtractive method compared to the Damascene process.

B. Device uniformity

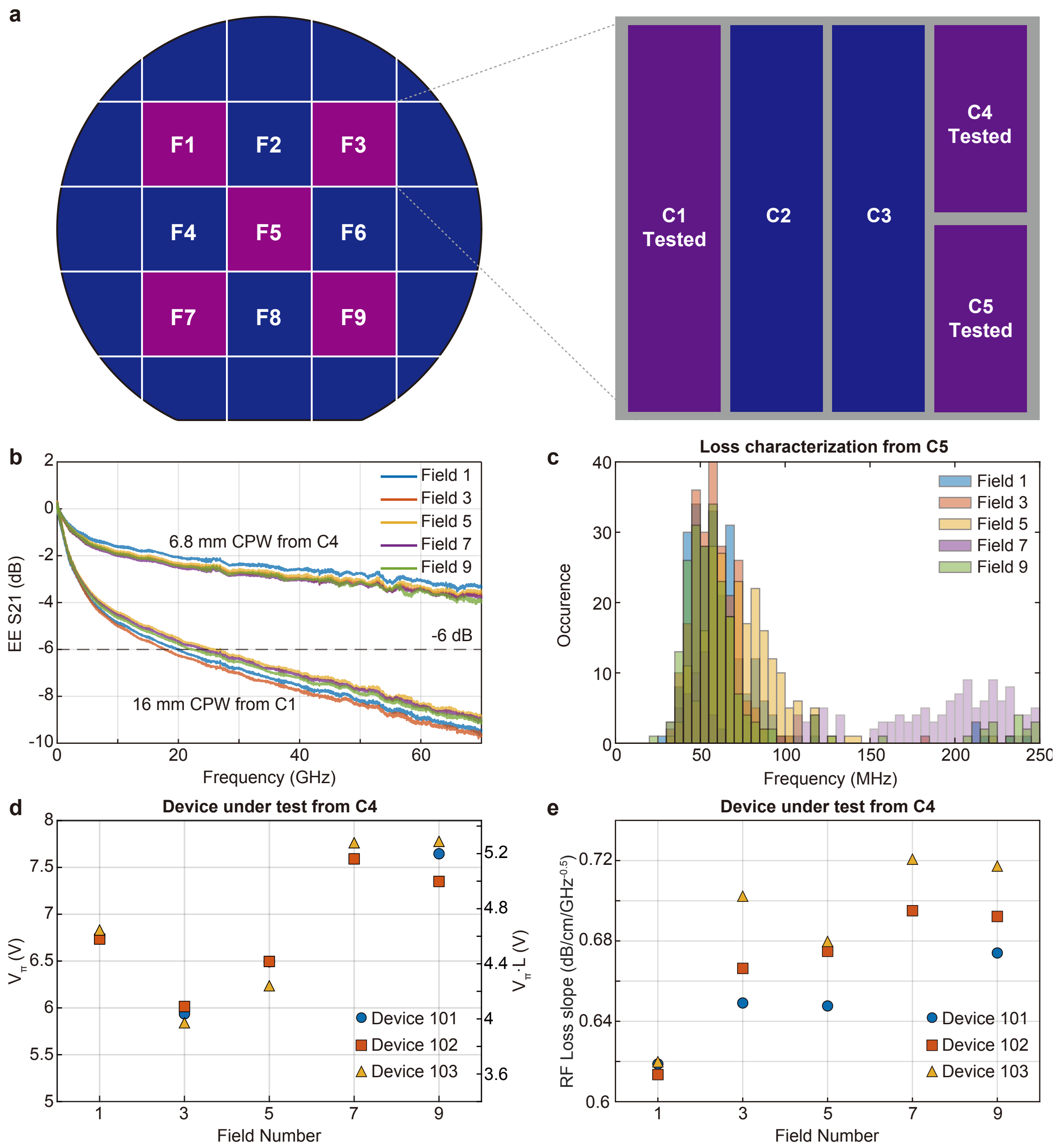

Supplementary Figure 5. **Wafer-scale characterization of EO modulators and microring resonators.** (a) Schematic showing the five fields selected for wafer-level testing (left panel) and the selected chips for inter-field device comparison (right panel). (b) Measured microwave electrical-to-electrical S_{21} response for 6.8 mm long and 16 mm long CPW electrodes, showing high uniformity across the five fields. (c) Intrinsic linewidth, $\kappa_0/2\pi$, histogram for microrings from the selected fields. All rings share the same design featuring a 112 GHz free spectral range. (d,e) Measured modulation efficiency and RF loss slope for three 6.8 mm MZMs within C4. The modulators share the same signal-ground gap yet have different signal electrode widths. Missing data points are due to device damage during handling or packaging.

As depicted in Supplementary Figure 5(a), device performance uniformity across the wafer is characterized by choosing five representative fields for testing as well as selecting three chips within a given field for evaluating inter-field device variations. From the obtained microwave transmission displayed in Supplementary Figure 5(b), the 3 dB EO bandwidth can be approximately calculated from the 6 dB electrical bandwidth when only considering the effects of microwave loss and assuming impedance matching and velocity matching [8, 9]. The electrical S_{21} curves for

both the 6.8 mm and 16 mm long devices are tightly grouped, indicating excellent consistency. Correspondingly, the estimated 3 dB EO bandwidth exceeds 67 GHz for the 6.8 mm modulators and is approximately 20 GHz for the 16 mm modulators across all tested fields. Optical loss uniformity was evaluated by measuring the intrinsic linewidth $\kappa_0/2\pi$ of microring resonators across the same five fields. Supplementary Figure 5(c) provides intrinsic linewidth histograms of these rings' resonances over a considered frequency range of 184-200 THz. The microrings are located on C5 and share the same design featuring a 112 GHz free-spectral range. Relevant statistics presented as **(Field number, Mean value, Median value, Max value)** are listed as follows: (F1, 66.8 MHz, 58.6 MHz, 45 MHz), (F3, 69.1 MHz, 57.7 MHz, 55 MHz), (F5, 77.8 MHz, 71.7 MHz, 55 MHz), (F7, 164 MHz, 187 MHz, 50 MHz), (F9, 85.6 MHz, 57.1 MHz, 55 MHz). Inter- and intra-field uniformity is highlighted in Supplementary Figure 5(d,e). The modulation efficiency and RF loss for three 6.8 mm long MZMs, with the same electrode gap and different signal widths (marked as Devices 101-103) in C4, demonstrate comparable performance to the reported device in the main text, with minor variations attributed to fabrication tolerances.

Regarding the insertion loss, Supplementary Table 2 details the insertion loss (IL) budgets for the 6.8 mm long single MZM devices. The "Taper-to-taper IL" represents the total fiber-to-chip coupling loss measured from a reference straight waveguide with two mode transition tapers, while the intrinsic "MZM IL" is obtained by subtracting the coupling loss from the "Total IL" of the modulator device. Missing data points in the table, such as F5, are due to some devices being damaged during handling or shipped away for high speed telecommunication testing. The MZM devices from the primary wafer exhibit a high IL near 20 dB as well as a total IL near 40 dB. This is attributed to a non-ideal DUV lithography exposure dose during the Si_3N_4 fabrication step, which resulted in malformed taper structures for both the edge couplers and the on-chip Y-splitters. To demonstrate the platform's intrinsic low-loss capability, we include data attributed to chips labeled as "Old sample" from our group's prior work. The devices feature identical optical designs with successfully patterned tapers. However, their high-frequency performance is uniformly degraded by parasitic capacitance loss, which we attribute to the high-power oxygen plasma used during the photoresist removal step. This sample shows a competitive coupling loss near 3.4 dB per facet and a reasonable on-chip MZM insertion loss of 5-8 dB, which confirms that the high loss on the current fabrication run is a correctable anomaly and not limited by optical design.

Supplementary Table 2. Insertion loss (IL) extracted for 6.8 mm MZMs.

Field	Taper-to-taper IL (dB)	Device number	Total IL (dB)	MZM IL (dB)
F1 (packaged)	/	Device 103	-23.0000	/ (packaged)
F3	-20.0736	Device 101	-35.5309	-15.4573
		Device 102	-29.6210	-9.5474
		Device 103	-25.904	-5.8304
F5	-26.3650	Device 101	-44.8847	-18.5197
		Device 102	-37.2224	-10.8574
		Device 103	-39.7224	-13.3574
F9	-25.7133	Device 101	-45.7785	-20.0652
		Device 102	-46.4975	-20.7842
		Device 103	-44.1042	-18.3909
Old sample	-6.8627	Device 101	-11.986	-5.1233
		Device 102	-11.5656	-4.7029
		Device 103	-14.6682	-7.8055

Supplementary Note 4. Simulations for tapered mode transitions

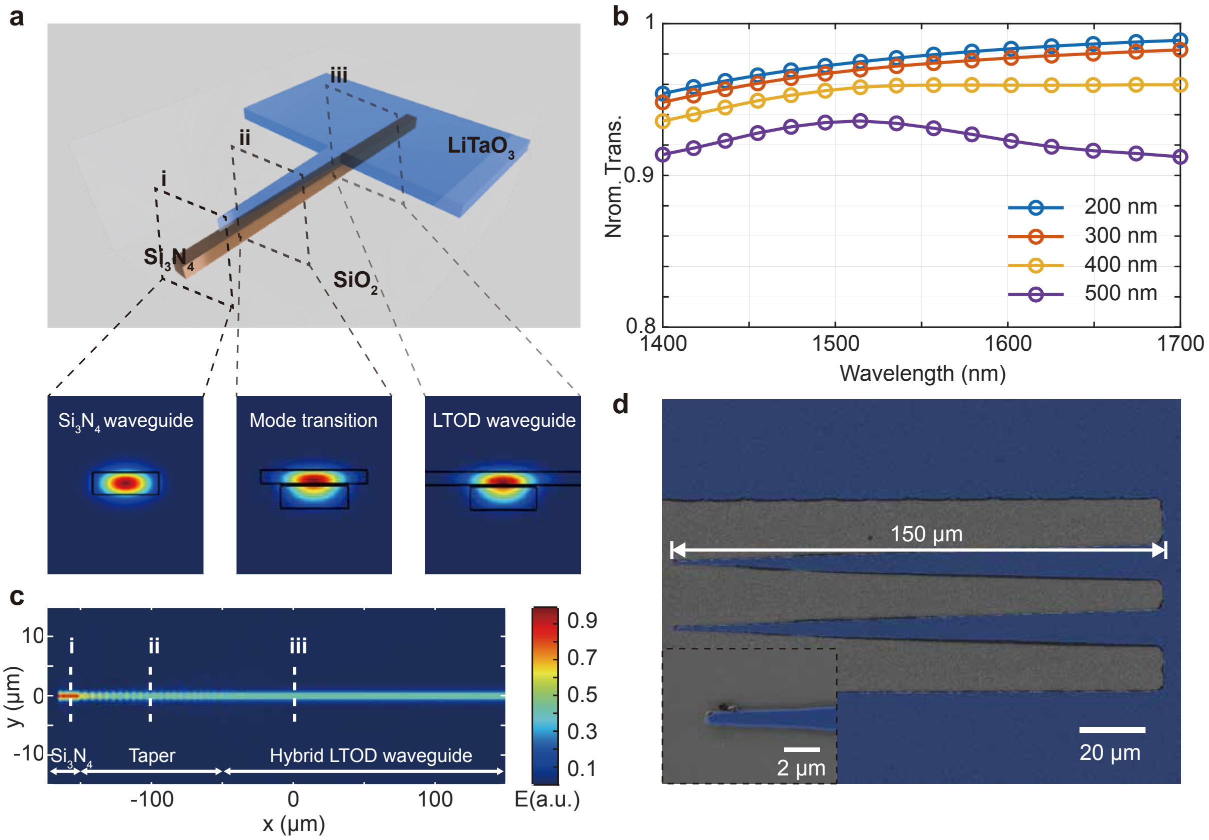

Supplementary Figure 6. Simulated transmission of silicon nitride-lithium tantalate waveguide transitions. (a) Schematic diagram and corresponding FDTD simulations of the adiabatic coupling from the Si_3N_4 waveguide to the hybrid Si_3N_4-$LiTaO_3$ (LTOD) waveguide. (b) Simulated transmission spectrum of the waveguide transition for various minimum $LiTaO_3$ taper widths. (c) Simulated electric field distribution of the adiabatic taper for a 500 nm minimum $LiTaO_3$ taper width. (d) Top-view scanning electron micrograph of the waveguide transition where the patterned $LiTaO_3$ layer features blue false colors.

Low-loss optical mode coupling is achieved using an adiabatic taper transition [10], enabling efficient single-mode transition from Si_3N_4 waveguides to hybrid Si_3N_4-$LiTaO_3$ waveguides (Supplementary Figure 6(a)). Based on the fabricated Si_3N_4-$LiTaO_3$ structure, the coupling design exhibits a 100 μm-long inverse taper and a 500 nm-wide tip, which are constructed in the etched $LiTaO_3$ film. As illustrated in Supplementary Figure 6(b,c), this tapered design facilitates high coupling efficiency across a broad wavelength range (1500 nm to 1640 nm) for Si_3N_4-$LiTaO_3$ modulators, achieving a low insertion loss of near 0.28 dB per facet. As shown in Supplementary Figure 6(c), the 500 nm minimal width of the taper primarily limits transmission. The patterning of the $LiTaO_3$ layer relied on a mask-less lithography method. As shown in the scanning electron micrograph of Supplementary Figure 6(d), this type of lithography produces coarser features resulting in greater insertion losses. The simulated data provided in Supplementary Figure 6(b) suggest that decreasing this width down to 200 nm can decrease the taper's insertion loss down to 0.08 dB. As previously demonstrated for heterogeneously integrated $LiNbO_3$-Si_3N_4 circuits, DUV optical lithography can reach these widths and insertion loss figures.

Supplementary Note 5. Microwave transmission and modulation efficiency

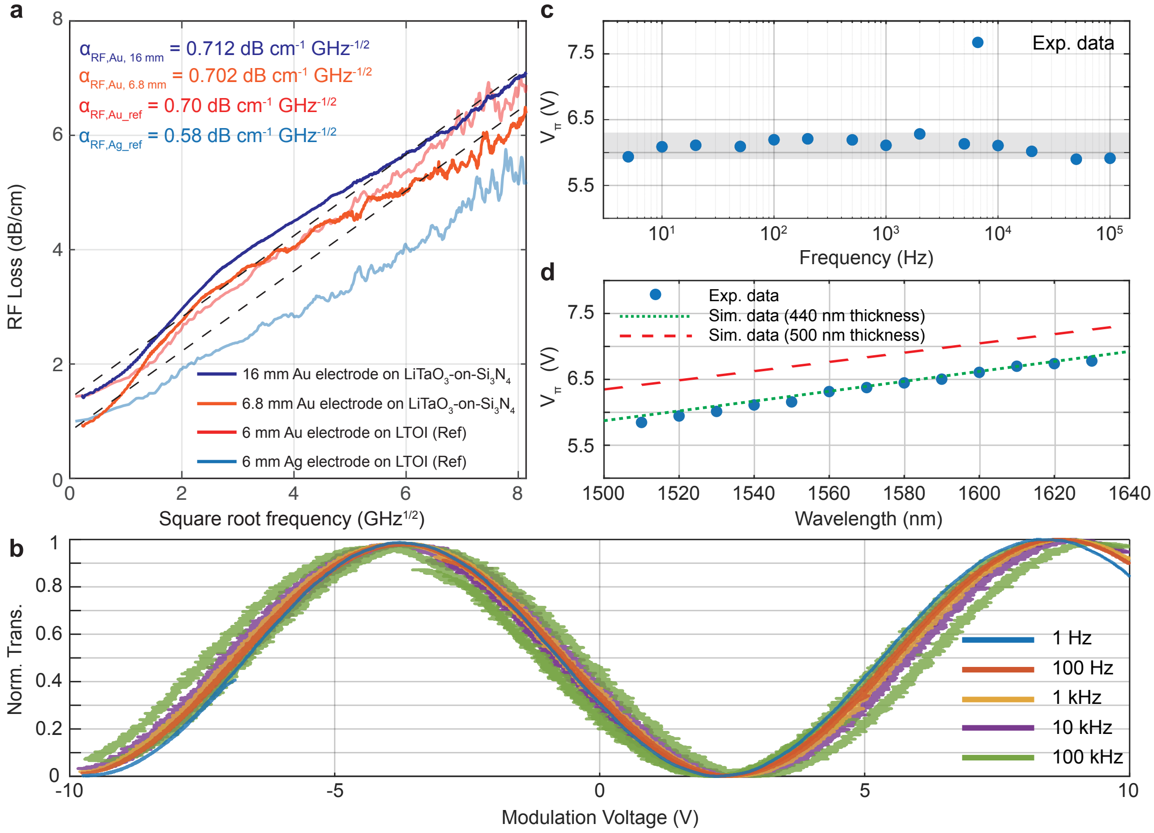

Supplementary Figure 7. **Modulator microwave transmission and modulation efficiency.** (a) Measured co-planar waveguide microwave losses on a square-root frequency axis, including the performance comparison with monolithic thin film $LiTaO_3$ modulators using 6 mm long gold electrodes and silver electrodes from Ref.[11]. (b) Measured transmission of the Si_3N_4-$LiTaO_3$ modulators driven by modulation frequencies from 1 Hz to 100 kHz. Extracted modulator V_π value at (c) different modulation frequencies at a fixed optical 1550 nm optical wavelength and (d) different optical wavelengths at a fixed 100 Hz modulation frequency.

We first characterized the RF attenuation properties of the fabricated CPW-type modulators. Supplementary Figure 7(a) presents the extracted microwave loss of the 6.8 mm long gold electrode on the Si_3N_4-$LiTaO_3$ platform, using a 67 GHz vector network analyzer (VNA). The square-root frequency dependence of RF loss (the black dashed line shown in Supplementary Figure 7(a)) indicates the dominant ohmic loss mechanism ($\alpha \propto \sqrt{f_{MW}}$), confirming that our fabrication techniques can effectively prevent parasitic-capacitance-induced loss [12, 13]. The measured RF attenuation is comparable to state-of-the-art ultrabroadband LTOI modulators with gold CPW electrodes [11]. Employing silver electrodes could further reduce ohmic losses, thus potentially enabling a higher EO bandwidth. However, this choice risks degrading the stability of the electrodes owing to silver's vulnerability to oxidation. Therefore, incorporating silver-based electrodes in Si_3N_4-$LiTaO_3$ modulators would require adapting them to wafer-scale fabrication and guaranteeing long-term device reliability.

To further demonstrate the dynamic performance of the Si_3N_4-$LiTaO_3$ Mach-Zehnder modulators, we investigated their EO performance under low-frequency RF modulation ranging from 1 Hz to 100 kHz (Supplementary Figure 7(b)). We extracted a stable half-wave voltage (V_π) with an average V_π value of 6.1 V from the experimental data. Supplementary Figure 7(c) shows that a thinner than expected Si_3N_4 waveguide can yield this lower value than the V_π anticipated from a 500 nm thickness. As shown in the wafer map of Supplementary Figure 4(a), such thickness variations can arise from uneven polishing stress distributions during the CMP step of the modulator's fabrication. Similarly, we studied the wavelength response of the modulator performance driven by a 100 Hz triangle wave signal (Supplementary Figure 7(d)), which features excellent agreement with our simulation results (red dashed line). This wavelength-dependent behavior is due to the increased optical mode expansion in the lithium tantalate thin film at longer wavelengths, leading to an enhanced microwave-optical field interaction.

Supplementary Note 6. Photonic package for DC-bias drift measurements

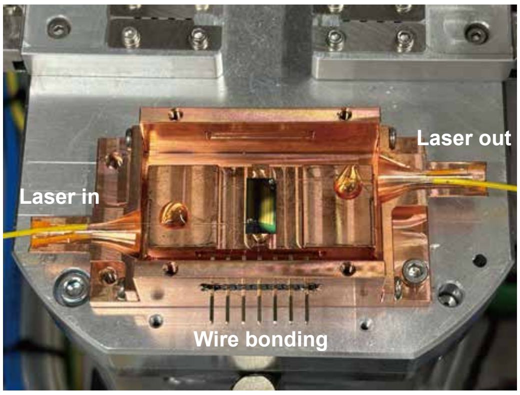

Supplementary Figure 8. **Photonic package for DC electro-optic stability measurements.** Image of the packaged Si_3N_4-$LiTaO_3$ modulator.

The DC bias drift of a modulator consists of the temporal variations in its optical output power while biased at a constant voltage (e.g., the quadrature point). These fluctuations typically arise from mechanisms such as charge relaxation and the photorefractive effect [14, 15]. Though the DC-stability of monolithic $LiTaO_3$ modulators was demonstrated in previous work [16], a similar study is still essential to verify whether this feature also applies to hybrid Si_3N_4-$LiTaO_3$ MZMs. To remove the influence of varying chip-to-fiber coupling efficiency, a robust and custom photonic package was engineered for this experiment. As shown in Supplementary Figure 8, the chip was first manually mounted onto a copper sub-mount with a UV-cure adhesive and then electrically connected with wire bonding (F&S Bondtec 56i). This step was followed by active alignment of the input/output fibers that were precisely maneuvered using multi-axis flexture stages, while the optical power was monitored with a photodiode. Once the coupling efficiency was optimized, the fibers were permanently fixed in place at the chip facets using high-precision epoxies.

Supplementary Note 7. Digital signal processing at the receiver for silicon nitride-lithium tantalate modulator-based communication experiments

In the data transmission experiments discussed in the main text, the optical signals were detected by photodiodes and the resulting photocurrents were digitized by a high-speed real-time oscilloscope (UXR 1004A, Keysight Technologies Inc.) operating at a sampling rate of 256 GSa/s with an analog bandwidth of 105 GHz. A total of 2^{23} samples corresponding to a time interval of approximately 33 µs were recorded. Offline, non-data-aided signal processing was employed to extract the transmitted data.

For both IMDD and coherent transmission schemes, the digitized signal was initially resampled to two samples per symbol. Timing recovery was then performed using a feedforward timing recovery algorithm, as described in [17] and [18], to estimate and correct the timing offset of the received signal. An adaptive receive filter, implemented as a time-domain linear equalizer, was subsequently applied.

In the IMDD case, linear Sato equalization [19] was utilized, followed by a linear post-equalizer based on the decision-directed least-mean-squares (DD-LMS) algorithm [20] to recover the transmitted data.

For coherent communications, a linear equalizer based on the constant modulus algorithm [21] was applied. This was followed by frequency offset compensation using a phase increment estimation algorithm [22], which corrects for the frequency mismatch between the optical carrier at the transmitter and the local oscillator at the receiver. Residual phase errors, originating from the phase noise of the transmitter and receiver lasers, was mitigated using the blind phase search algorithm [23]. Finally, a DD-LMS-based linear post-equalizer [20] was employed to recover the complex-valued transmitted symbols.

Supplementary Note 8. Extended signal quality and energy efficiency analysis for the high-speed data transmission experiments

This section provides additional details regarding the signal quality of the intensity-modulation and direct-detection (IMDD) and coherent data transmission results presented in the main manuscript, comprising the normalized generalized mutual information (NGMI) for both transmission experiments and the error vector magnitude (EVM) for quadrature phase-shift keying (QPSK) and 16-state quadrature amplitude modulation (16QAM) transmission, see Supplementary Figure 9(c). Furthermore, the electrical energy dissipation per transmitted bit is estimated for different modulation formats and symbol rates.

A. Normalized generalized mutual information (NGMI) and error vector magnitude (EVM)

The generalized mutual information (GMI) is directly related to the achievable information rate (AIR) for bit-interleaved coded modulation and is commonly used to define performance prediction thresholds, especially for soft-decision forward error correction (SD-FEC) schemes [24]. We derive the GMI of the transmitted signals from the log-likelihood ratios (LLRs) of the received symbols based on a linear channel model with additive white Gaussian noise (AWGN) as the only impairment [25]. To compare different modulation formats, we calculate the NGMI by normalizing the GMI by the number of bits that can be encoded into a single symbol. Supplementary Figure 9(a) shows the NGMI for four-level pulse-amplitude modulation (PAM4) signals transmitted at different symbol rates in our IMDD experiment. The NGMI for QPSK and 16QAM signals transmitted in our coherent transmission experiment are depicted in Supplementary Figure 9(b). Based on these values, we select suitable forward error correction (FEC) codes with the nearest lower NGMI threshold as given in [26, Table 2] and evaluate the net data rate (NDR) by multiplying the line rates and the associated FEC code rates, see Figure 3(c) and Figure 4(d) of the main manuscript. As the symbol rate increases, the signal-to-noise-and-distortion ratio (SNDR) of the received signal decreases, and FEC implementations with larger overheads are required to enable error-free decoding. As a consequence, we observe a reduction of the NDR beyond 192 GBd for PAM4 signals, see Figure 3(c) of the main manuscript.

The EVM is an alternative metric of the quality of the received signal in coherent transmission systems and quantifies the root-mean-square (RMS) distance between the received complex modulation symbols E_r and their ideal positions in the constellation diagram E_t [27]. We calculate the EVM normalized to the average power as

$$\mathrm{EVM} = \sqrt{\frac{\sum_{i=1}^{N} |E_{\mathrm{r},i} - E_{\mathrm{t},i}|^2}{\sum_{i=1}^{N} |E_{\mathrm{t},i}|^2}}, \tag{7}$$

where N is the number of symbols over which the EVM is evaluated. Supplementary Figure 9(c) shows the resulting EVM for QPSK and 16QAM symbols after equalization as a function of the symbol rate. As expected, the EVM increases as the signal quality decreases for larger symbol rates.

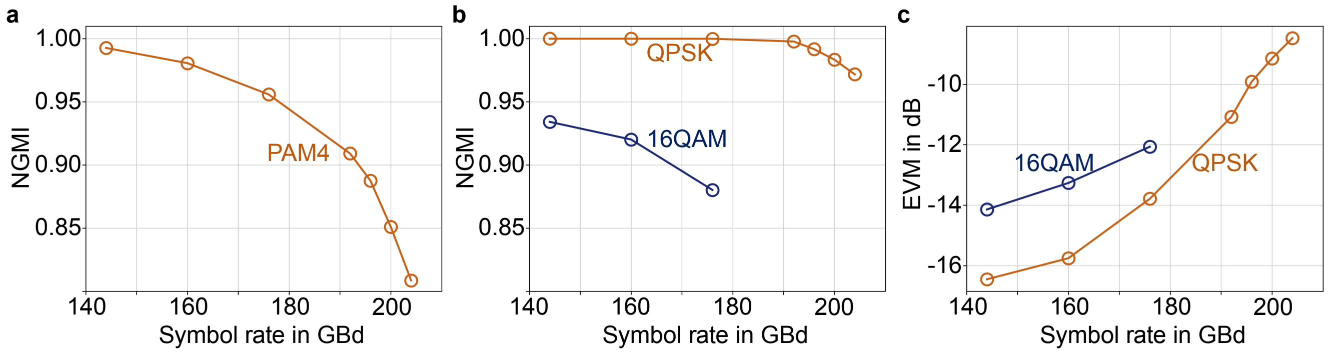

Supplementary Figure 9. Extended signal quality analysis for high-speed data transmission experiments. (a) Normalized generalized mutual information (NGMI) for four-level pulse-amplitude modulation (PAM4) signals received in our intensity-modulation and direct-detection (IMDD) experiment. (b) NGMI for quadrature phase-shift keying (QPSK, brown) and 16-state quadrature-amplitude modulation (16QAM, blue) signals received in our coherent transmission experiment. All signals were transmitted using Si_3N_4-$LiTaO_3$ modulators across various symbol rates. (c) Error vector magnitude (EVM) of the QPSK (brown) and 16QAM (blue) symbols extracted at the receiver for different symbol rates.

B. Energy dissipation

To estimate the electrical energy dissipation per transmitted bit, we first need to extract the voltage levels that were fed to the modulator during the transmission experiment. To this end, the electrical transmitter was directly connected to the real-time oscilloscope (RTO, UXR 1004A, Keysight Technologies Inc.) and operated with the same parameters and signals as in the optical transmission experiment.

In the case of the IMDD experiment, the transmitter consisted of a high-speed arbitrary-waveform generator (AWG, M8199B, Keysight Technologies Inc.) and a subsequent RF amplifier (SHF T850 B, SHF Communication Technologies AG), which was directly connected to the RF probe during the transmission experiment. The AWG and the RF amplifier are connected by a 20 cm-long RF cable. For measuring the voltage levels that were fed to the modulator during the transmission experiment, we connect the output of the RF amplifier directly to the RTO. To estimate the electrical energy dissipation during the data transmission experiment, we calculated the average electrical power fed to the modulator from the voltage samples recorded by the RTO. Assuming impedance matching between the terminated modulator and the internal resistance of the transmitter ($R = 50\,\Omega$), the average electrical power is calculated from the instantaneous voltage $u(t)$ by

$$\overline{P} = \frac{1}{T}\int_{t=0}^{T} \frac{u(t)^2}{R}\mathrm{d}t\ , \tag{8}$$

where T is an observation time that covers many symbols ($T = 32.8\,\mu\mathrm{s}$ in our experiments). The electrical energy dissipation per transmitted payload bit is given by dividing the average electric power by the NDR

$$W_{\mathrm{bit}} = \frac{\overline{P}}{\mathrm{NDR}}\ . \tag{9}$$

Note that the transmission experiments at the various symbol rates were conducted with a constant drive-voltage swing applied to the MZM, which was ensured by re-adjusting the output voltage settings of the AWG for each symbol rate as to maintain the voltage difference between the highest and the lowest PAM4 signal level in the range (1.25 ± 0.05) V. Supplementary Figure 10(a) shows the net energy per bit for transmitted PAM4 signals at various symbol rates. Since the drive voltages were kept constant, the energy dissipation follows the trend of the inverse NDR, see Figure 3(c) of the main manuscript. The lowest energy dissipation per payload bit amounts to $12.9\,\mathrm{fJ\,bit^{-1}}$, reached at a symbol rate of 176 GBd. Non-ideal voltage settings lead to smaller deviations from of the measured values from a continuous trend, as indicated by a dashed line in Supplementary Figure 10(a).

For the coherent data transmission experiments, the drive voltages applied to both MZMs must be considered individually, since the MZM in our sample device turned out to have different half-wave voltages. Due to the limited physical space between the two input connectors at the feeding RF-probe, the electrical transmitter configuration differs from that one used in the IMDD setup. Specifically, two outputs of the AWG were each directly connected to separate broadband RF amplifiers, followed by two broadband bias-tees and two 20 cm-long RF cables. The components in both signal paths are nominally identical. In this experiment, we kept the voltage settings of the AWG constant, leading to a decrease of the voltage swings coupled to the MZM with increasing symbol rates. As in the IMDD case, the voltage levels for the transmitted QPSK and 16QAM symbols are extracted by connecting the RF cables to the input of the RTO, and the average electrical power and the electrical energy dissipation per transmitted payload bit were estimated in analogy to Equations (8) and (9). For the MZM transmitting the in-phase signal, the drive voltage difference between the lowest and the highest signal level decreases from 0.8 V at 144 GBd to 0.4 V at 204 GBd. For the quadrature MZM, the voltage swing reduced from 1.6 V to 0.8 V over the same symbol rate range. The electrical energy per transmitted payload bit is depicted in Supplementary Figure 10(b). The minimum net electrical energy dissipation per bit amounts to $9.4\,\mathrm{fJ\,bit^{-1}}$ and was achieved for 16QAM signals with a symbol rate of 176 GBd.

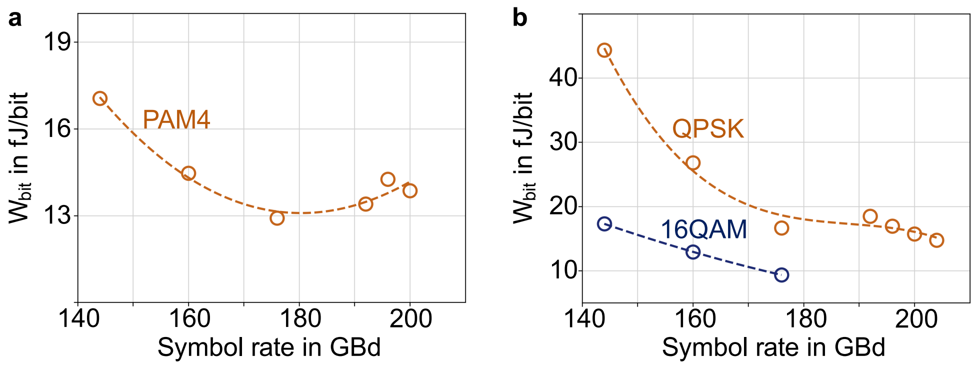

Supplementary Figure 10. **Electrical energy efficiency analysis for high-speed data transmission experiments.** (a) Estimated electrical energy dissipation per payload bit calculated from the drive voltages measured for four-level pulse-amplitude modulation (PAM4) signals using intensity-modulation and direct-detection (IMDD) and (b) for quadrature phase-shift keying (QPSK, brown) and 16-state quadrature-amplitude modulation (16QAM, blue) signals. A third-order polynomial fit (dashed line) provides a guide to the eye. All quantities are plotted for different symbol rates. Please note the different scales of the vertical axes.

Supplementary Note 9. Comparison to the state-of-the-art

To benchmark our device against the state-of-the-art, we summarize the performance metrics of leading EO modulator technologies in Supplementary Table 3. These technologies include monolithic platforms such as $LiNbO_3$-on-insulator (LNOI), $LiTaO_3$-on-insulator (LTOI), $BaTiO_3$-on-insulator (BTOOI), as well as heterogeneous photonic platforms based on $BaTiO_3$-Si_3N_4, $LiNbO_3$-Si_3N_4 and $LiTaO_3$-Si_3N_4. Our devices exhibit a compelling performance profile for both single MZM and IQ modulator architectures by achieving superior data throughput while maintaining competitive modulation efficiency.

Supplementary Table 3. **Performance metrics of high-speed EO modulators.**

**Ref.**	**Platform**	**Type**	**BW**[a] **[GHz]**	$V_\pi \cdot L$ [V · cm]	IL_{total} **[dB]**	**Format**	**LR**[b] **[Gbit/s]**	**BER**
[28]	LNOI	single MZM	> 45	2.8	NA	PAM 4	140	2.10×10^{-5}
		single MZM	> 45	2.8	NA	PAM 8	210	1.50×10^{-2}
		single MZM	~ 100	2.2	NA	NA	NA	NA
[29]	LNOI	single MZM	~ 170	3.3	16	PAM 4	200	5.50×10^{-3}
		single MZM	~ 170	3.3	16	PAM 8	240	1.10×10^{-2}
[30]	LNOI	IQ	48	2.47	8.6	QPSK	220	8.63×10^{-6}
		IQ	48	2.47	8.6	16 QAM	320	8.41×10^{-3}
[31]	LNOI	IQ	110	2.3	12.9	DP 16 QAM[b]	1600	NA
		IQ	110	2.3	12.9	DP 64 QAM[b]	2220	NA
[11]	LTOI	single MZM	~ 110	2.88	14.00	PAM 8	528	3.80×10^{-2}
[32]	BTOOI	single MZM	NA	2.32	NA	NA	NA	NA
[33]	$BaTiO_3$-Si_3N_4	single MZM	NA	0.48	12	PAM 4	212	3.50×10^{-3}
[34]	$LiTaO_3$-Si_3N_4	single MZM	~ 70	2.3	NA	PAM 4	320	$\sim 1.60 \times 10^{-2}$
[35]	$LiNbO_3$-Si_3N_4	single MZM	~ 110	3.4	5.8	NA	NA	NA
This work	$LiTaO_3$-Si_3N_4	single MZM	100	4.08	11.56	PAM 4	400	3.00×10^{-2}
		IQ	34	4.05	NA	QPSK	408	7.00×10^{-3}
		IQ	34	4.05	NA	16 QAM	704	3.08×10^{-2}

NA: not available.
[a]BW: Bandwidth.
[b]LR: Line rate.
[c]DP: Dual-polarization.

Supplementary References

[1] G. Besnard, B.-Y. Nguyen, and C. Maleville, Smart cut™ technology: from substrate enginnering to advanced 3d integration, in *2022 International Conference on IC Design and Technology (ICICDT)* (2022) pp. 81–83.
[2] X.-Q. Feng and Y. Huang, Mechanics of smart-cut® technology, Int. J. Solids Struct. **41**, 4299 (2004).
[3] S. G. Johnson, M. Ibanescu, M. Skorobogatiy, O. Weisberg, J. Joannopoulos, and Y. Fink, Perturbation theory for maxwell's equations with shifting material boundaries, Phys. Rev. E **65**, 066611 (2002).
[4] H. Larocque, D. L. P. Vitullo, A. Sludds, H. Sattari, I. Christen, G. Choong, I. Prieto, J. Leo, H. Zarebidaki, S. Lohani, B. T. Kirby, Ö. Soykal, M. Soltani, A. H. Ghadimi, D. R. Englund, and M. Heuck, Photonic crystal cavity iq modulators in thin-film lithium niobate, ACS Photonics **11**, 3860 (2024).
[5] Y. Liu, H. Li, J. Liu, S. Tan, Q. Lu, and W. Guo, Low V_π thin-film lithium niobate modulator fabricated with photolithography, Opt. Express **29**, 6320 (2021).
[6] Y. Hu, D. Zhu, S. Lu, X. Zhu, Y. Song, D. Renaud, D. Assumpcao, R. Cheng, C. J. Xin, M. Yeh, H. Warner, X. Guo, A. Shams-Ansari, D. Barton, N. Sinclair, and M. Loncar, Integrated electro-optics on thin-film lithium niobate, Nat. Rev. Phys. **7**, 237 (2025).
[7] X. Ji, R. N. Wang, Y. Liu, J. Riemensberger, Z. Qiu, and T. J. Kippenberg, Efficient mass manufacturing of high-density, ultra-low-loss Si3N4 photonic integrated circuits, Optica **11**, 1397 (2024).
[8] G. Ghione, Modulators, in *Semiconductor Devices for High-Speed Optoelectronics* (Cambridge University Press, 2009) p. 356–439.
[9] D. Zhu, L. Shao, M. Yu, R. Cheng, B. Desiatov, C. J. Xin, Y. Hu, J. Holzgrafe, S. Ghosh, A. Shams-Ansari, E. Puma, N. Sinclair, C. Reimer, M. Zhang, and M. Lončar, Integrated photonics on thin-film lithium niobate, Adv. Opt. Photon. **13**, 242 (2021).
[10] M. Churaev, R. N. Wang, A. Riedhauser, V. Snigirev, T. Blésin, C. Möhl, M. H. Anderson, A. Siddharth, Y. Popoff, U. Drechsler, D. Caimi, S. Hönl, J. Riemensberger, J. Liu, P. Seidler, and T. J. Kippenberg, A heterogeneously integrated lithium niobate-on-silicon nitride photonic platform, Nat. Commun. **14**, 3499 (2023).
[11] C. Wang, D. Fang, J. Zhang, A. Kotz, G. Lihachev, M. Churaev, Z. Li, A. Schwarzenberger, X. Ou, C. Koos, and T. J. Kippenberg, Ultrabroadband thin-film lithium tantalate modulator for high-speed communications, Optica **11**, 1614 (2024).
[12] M. Shen, L. Yang, Y. Xu, and H. X. Tang, Parasitic conduction loss of lithium niobate on insulator platform, Appl. Phys. Lett. **124**, 101107 (2024).
[13] P. Yang, S. Sun, Y. Zhang, R. Cao, H. He, H. Xue, and F. Liu, High-bandwidth lumped mach-zehnder modulators based on thin-film lithium niobate, Photonics **11**, 399 (2024).
[14] S. Yamada and M. Minakata, Dc drift phenomena in LiNbO3 optical waveguide devices, Jpn. J. Appl. Phys **20**, 733 (1981).
[15] J. P. Salvestrini, L. Guilbert, M. Fontana, M. Abarkan, and S. Gille, Analysis and control of the dc drift in $LiNbO_3$-based mach–zehnder modulators, J. Light. Technol. **29**, 1522 (2011).
[16] H. Wang, X. Xing, Z. Ruan, J. Yu, K. Chen, X. Ou, and L. Liu, Optical switch with an ultralow dc drift based on thin-film lithium tantalate, Opt. Lett. **49**, 5019 (2024).
[17] S. Barton and Y. Al-Jalili, A symbol timing recovery scheme based on spectral redundancy, in *IEEE Colloquium on Advanced Modulation and Coding Techniques for Satellite Communications* (1992) pp. 3/1–3/6.
[18] P. Matalla, M. S. Mahmud, C. Füllner, C. Koos, W. Freude, and S. Randel, Hardware comparison of feed-forward clock recovery algorithms for optical communications, in *OFC 2021*, Th1A.10 (2021).
[19] Y. Sato, A method of self-recovering equalization for multilevel amplitude-modulation systems, IEEE Trans. Commun. **23**, 679 (1975).
[20] S. Randel, D. Pilori, S. Corteselli, G. Raybon, A. Adamiecki, A. Gnauck, S. Chandrasekhar, P. Winzer, L. Altenhain, A. Bielik, and R. Schmid, All-electronic flexibly programmable 864-Gb/s single-carrier PDM-64-QAM, in *OFC 2014*, Th5C.8 (2014).
[21] S. Moshirian, S. Ghadami, and M. Havaei, Blind channel equalization, Preprint at http://arxiv.org/abs/1208.2205 (2012).
[22] A. Leven, N. Kaneda, U.-V. Koc, and Y.-K. Chen, Frequency estimation in intradyne reception, IEEE Photon. Technol. Lett. **19**, 366 (2007).
[23] T. Pfau, S. Hoffmann, and R. Noe, Hardware-efficient coherent digital receiver concept with feedforward carrier recovery for m -QAM constellations, J. Light. Technol. **27**, 989 (2009).
[24] L. Schmalen, Performance metrics for communication systems with forward error correction, in *2018 European Conference on Optical Communication (ECOC)* (2018) pp. 1–3.
[25] M. Ivanov, C. Häger, F. Brännström, A. Graell i Amat, A. Alvarado, and E. Agrell, On the information loss of the max-log approximation in BICM systems, IEEE Transactions on Information Theory **62**, 3011 (2016).
[26] Q. Hu, R. Borkowski, Y. Lefevre, J. Cho, F. Buchali, R. Bonk, K. Schuh, E. De Leo, P. Habegger, M. Destraz, N. Del Medico, H. Duran, V. Tedaldi, C. Funck, Y. Fedoryshyn, J. Leuthold, W. Heni, B. Baeuerle, and C. Hoessbacher, Ultrahigh-net-bitrate 363 Gbit/s PAM-8 and 279 Gbit/s polybinary optical transmission using plasmonic mach-zehnder modulator, Journal of Lightwave Technology **40**, 3338 (2022).
[27] R. Schmogrow, B. Nebendahl, M. Winter, A. Josten, D. Hillerkuss, S. Koenig, J. Meyer, M. Dreschmann, M. Huebner, C. Koos, J. Becker, W. Freude, and J. Leuthold, Error vector magnitude as a performance measure for advanced modulation formats, IEEE Photonics Technology Letters **24**, 61 (2012).

[28] C. Wang, M. Zhang, X. Chen, M. Bertrand, A. Shams-Ansari, S. Chandrasekhar, P. Winzer, and M. Lončar, Integrated lithium niobate electro-optic modulators operating at cmos-compatible voltages, Nature **562**, 101 (2018).

[29] F. Arab Juneghani, M. Gholipour Vazimali, J. Zhao, X. Chen, S. T. Le, H. Chen, E. Ordouie, N. K. Fontaine, and S. Fathpour, Thin-film lithium niobate optical modulators with an extrapolated bandwidth of 170 ghz, Adv. Photonics **4**, 2200216 (2023).

[30] M. Xu, M. He, H. Zhang, J. Jian, Y. Pan, X. Liu, L. Chen, X. Meng, H. Chen, Z. Li, X. Xiao, S. Yu, S. Yu, and X. Cai, High-performance coherent optical modulators based on thin-film lithium niobate platform, Nat. Commun. **11**, 3911 (2020).

[31] S. Almonacil, H. Mardoyan, F. Jorge, F. Pittalà, M. Xu, B. Krueger, F. Blache, B. Duval, L. Chen, Y. Yan, X. Ye, A. Ghazisaeidi, S. Rimpf, Y. Zhu, J. Wang, M. Goix, Z. Hu, M. Duthoit, M. Gruen, X. Cai, and J. Renaudier, 260-gbaud single-wavelength coherent transmission over 100-km ssmf based on novel arbitrary waveform generator and thin-film niobate i/q modulator, Journal of Lightwave Technology **41**, 3674 (2023).

[32] Z. Dong, A. Raju, A. B. Posadas, M. Reynaud, A. A. Demkov, and D. M. Wasserman, Monolithic barium titanate modulators on silicon-on-insulator substrates, ACS Photonics **10**, 4367 (2023).

[33] F. Eltes, W. Li, E. Berikaa, M. Samiul Alam, S. Bernal, C. Minkenberg, D. V. Plant, and S. Abel, Thin-film bto-based modulators enabling 200 gb/s data rates with sub 1 vpp drive signal, in *2023 Optical Fiber Communications Conference and Exhibition (OFC)* (2023) pp. 1–3.

[34] M. Niels, T. Vanackere, E. Vissers, T. Zhai, P. Nenezic, J. Declercq, C. Bruynsteen, S. Niu, A. Moerman, O. Caytan, *et al.*, A high-speed heterogeneous lithium tantalate silicon photonics platform, Preprint at https://arxiv.org/abs/2503.10557 (2025).

[35] M. A. Rahman, F. Valdez, V. Mere, C. O. de Beeck, P. Wuytens, and S. Mookherjea, High-performance hybrid lithium niobate electro-optic modulators integrated with low-loss silicon nitride waveguides on a wafer-scale silicon photonics platform, Preprint at https://arxiv.org/abs/2504.00311 (2025).
